# Draft Genome Sequence of *Mycobacterium fortuitum* Isolated from Murine Brain

**DOI:** 10.1128/genomeA.00191-16

**Published:** 2016-03-31

**Authors:** Alok Kumar Singh, Pratiksha Karaulia, Sidharth Chopra, Arunava Dasgupta

**Affiliations:** Division of Microbiology, CSIR-Central Drug Research Institute, Lucknow, Uttar Pradesh, India

## Abstract

*Mycobacterium fortuitum* subsp. *fortuitum* ATCC 6841 is a type and standard laboratory testing quality control strain. We report here the completed draft genome sequence for a strain isolated from the brains of *M. fortuitum*-infected mice.

## GENOME ANNOUNCEMENT

*Mycobacterium fortuitum* is a fast-growing nontuberculous opportunistic human pathogen causing a wide variety of infections ranging from pulmonary to joint and trauma infections. It is a significant nosocomial problem, as it is frequently the causative agent of infections in surgically implanted devices, such as catheters, and other skin and soft tissue infections. It is extensively distributed in the natural environment, including in water, sewage, and dirt (1). Although it is generally drug susceptible, its treatment is complicated by inherent drug resistance to most of the antituberculosis drugs, combined with poor diagnostic guidelines, thus necessitating the utilization of multiple oral agents, such as carbapenems, fluoroquinolones, and macrolides, with usually varied prognosis (2).

Apart from other clinical infestations, *M. fortuitum* has also been known to cause cerebral infections (3). In order to better understand the host-pathogen dynamics in nontuberculous mycobacterial cerebral infections, we established a mouse model for brain infection utilizing *M. fortuitum* subsp. *fortuitum* ATCC 6841. In order to determine whether there are any genetic changes associated with cerebral infections, we isolated *M. fortuitum* from the brains of infected mice and performed whole-genome sequencing.

*M. fortuitum* ATCC 6841 is a clinical isolate, isolated from a patient with cold abscess, and it is a type and standard laboratory testing quality control strain. *M. fortuitum* was purchased from the American Type Culture Collection (ATCC), grown at 37°C in Middlebrook 7H9 broth supplemented with 10% albumin-dextrose-catalase (Becton, Dickinson) for 48 h, and genomic DNA was extracted using standard protocols.

The genomic DNA was sequenced on the Ion Torrent PGM system (Life Technologies), according to the manufacturer’s protocols for 200-bp genomic DNA (gDNA) fragment library preparation (IonXpress Plus gDNA and amplicon library preparation), template preparation (Ion 200 Template OT2 preparation kit), and sequencing (Ion PGM 200 sequencing kit version 2). A total of 527 Mb of data were obtained, with an accumulated length of 6,554,407 bp (30× fold coverage). A draft reference assembly was generated by mapping reads to the annotated reference genome of *M. fortuitum* accession no. CP011269. The annotation was transferred from the reference genome to the new draft assembly. All computational analysis was performed using CLC bio Genomics Workbench version 8.5. A total of 8,006 open reading frames (ORFs) were predicted, out of which 4,959 (61%) ORFs had an assigned function, 2,414 (30%) ORFs were conserved hypothetical, and the remaining 1,647 (20%) ORFs were classified as either of unknown function or hypothetical proteins, with 298 tRNAs and 34 rRNAs.

Detailed genomic data analysis from this draft genome assembly is under way to obtain a finer resolution and comparative genomics to decipher mutations that might play an important role in cerebral infections caused by *M. fortuitum*.

### Nucleotide sequence accession number.

This whole-genome project has been deposited at the DDBJ/EMBL/GenBank under the accession no. CP014258.
